# AICAR-Induced AMPK Activation Inhibits the Noncanonical NF-κB Pathway to Attenuate Liver Injury and Fibrosis in BDL Rats

**DOI:** 10.1155/2018/6181432

**Published:** 2018-12-19

**Authors:** Haoyang Zhu, Yichao Chai, Dinghui Dong, Nana Zhang, Wenyan Liu, Tao Ma, Rongqian Wu, Yi Lv, Liangshuo Hu

**Affiliations:** ^1^Department of Hepatobiliary Surgery, First Affiliated Hospital, Xi'an Jiaotong University, Xi'an 710061, Shaanxi Province, China; ^2^National Local Joint Engineering Research Center for Precision Surgery & Regenerative Medicine, Xi'an 710061, Shaanxi Province, China; ^3^Institute for Cancer Research School of Basic Medical Science of Xi'an Jiaotong University, Xi'an 710061, Shaanxi Province, China

## Abstract

**Background:**

To evaluate the AMP-activated protein kinase- (AMPK-) mediated signaling and NF-*κ*B-related inflammatory pathways that contribute to cholestatic diseases in the bile duct ligation (BDL) rat model of chronic cholestasis and verify the protective role of 5-Aminoimidazole-4-carboxamide1-*β*-D-ribofuranoside (AICAR) against hepatic injury and fibrosis triggered by cholestasis-related inflammation.

**Methods:**

Animals were randomly divided into three groups: sham-operated group, BDL group, and BDL+ AICAR group. Cholestatic liver injury was induced by common BDL. Two weeks later, rats in BDL+AICAR group started receiving AICAR treatment. Hepatic pathology was examined by haematoxylin and eosin (H&E) and sirius red staining and hydroxyproline assay was performed in evaluating the severity of hepatic cirrhosis. Real-time PCR and Western blot were performed for RNA gene expression of RNA and protein levels, respectively.

**Results:**

The BDL group showed liver injury as evidenced by histological changes and elevation in serum biochemicals, ductular reaction, fibrosis, and inflammation. The mRNA expression of canonical NF-*κ*B inflammatory cytokines such as TNF-*α*, IL-1*β*, TGF-*β*, and the protein of noncanonical NF-*κ*B, P100, and P52 was upregulated in the livers of BDL rats. The BDL rats with the administration of AICAR could induce AMPK activation inhibiting the noncanonical NF-*κ*B pathway to attenuate liver injury and fibrosis in BDL rats.

**Conclusion:**

The BDL model of hepatic cholestatic injury resulting in activation of Kupffer cells and recruitment of immune cells might initiate an inflammatory response through activation of the NF-*κ*B pathway. The AMPK activator AICAR significantly alleviated BDL-induced inflammation in rats by mainly inhibiting the noncanonical NF-*κ*B pathway and thus protecting against hepatic injury and fibrosis triggered by BDL.

## 1. Introduction

Cholestasis is defined as a reduction in bile flow due to intra- or extrahepatic bile duct obstruction or impaired hepatocyte secretion. This occurs in a series of chronic liver diseases, such as primary biliary cirrhosis (PBC) [1] and primary sclerosing cholangitis (PSC) [2], which leads to liver dysfunction, fibrosis, and ultimately liver failure. These cholestatic diseases are difficult to treat effectively and transplantation is required in advanced stage with portal hypertension or liver failure [3].

Bile duct ligation (BDL) is an experimental animal model for extrahepatic bile duct obstruction that entails the ramification of small bile ducts within liver tissue accompanied by leukocytes infiltration and subsequent fibrosis [4]. The BDL model is widely used in experimental studies of cholestatic diseases [5].

Early studies of chronic cholestatic disorders were focused on bile-acid-induced hepatocellular injury and apoptosis [6]. Recent works highlighted the role of inflammation in the pathophysiology of these diseases. Inflammation caused by the retention of bile acids is believed to play an important role in the progression to end-stage liver diseases [7]. Cholangiocytes are the epithelial cells of bile ducts that can secrete cytokines and chemokines and subsequently activate Kupffer cells and recruit immune cells to inflamed liver tissue [8]. It is also known that. during the chronic inflammation, proliferation of cholangiocytes and activated hepatic stellate cells contributes to the initiation and progression of liver fibrosis [9]. However, the molecular mechanisms and pathological changes of these diseases are not clear yet.

Nuclear factor kappa beta (NF-*κ*B) is the main regulator of oxidative stress and inflammatory response [10]. It plays a central regulatory role in the expression of various cytokines involved in the occurrence of liver fibrosis [11]. NF-*κ*B is maintained in an inactive state in the cytosol, but different stimuli can induce I*κ*B phosphorylation [12]. Active NF-*κ*B can subsequently be translocated from the cytosol to the nucleus where it activates the transcription of genes, including IL-6, TNF-*α*, IL-1*β*, and iNOS, which are involved in the inflammatory response and lead to cell injury. AMP-activated protein kinase (AMPK), an upstream protein of NF-*κ*B, is a critical signaling macromolecule and recognized as a key cellular metabolic sensor for maintaining the levels of ADP/AMP/ATP [13]. It has been reported that the activated AMPK could inhibit NF-*κ*B signaling through its downstream target molecules such as SIRT1, Forkhead box O (FOXO), and the peroxisome proliferator-activated receptor coactivator -1*α*(PGC-1*α*) and subsequently reduce the expression of inflammatory factors [14]. Recently, AMPK has attracted the attention of researchers for its potential role in cell signaling and the regulation of cell polarity, fibrosis, and bile acid homeostasis in cholestatic liver diseases [15]. The 5-Aminoimidazole-4-carboxamide1-*β*-D-ribofuranoside (AICAR) is an AMP analogue, which could activate AMPK and increase the AMP/ATP ratio in the cell and mimics. To our knowledge, there have been few reports on the effects of long-term administration of AMPK-agonist AICAR on liver inflammation induced by chronic cholestasis [16]. Additionally, the mitogen-activated protein kinase (MAPK) signaling pathway is a ubiquitous and important signaling pathway in eukaryotic cells that regulates cell proliferation, differentiation, apoptosis, and stress response and has been reported to be closely related to the activation of the NF-*κ*B pathway [17].

In this study, we analysed the changes of inflammatory cytokines in the livers of BDL rats to determine the contribution of the BDL-triggered inflammation in liver fibrosis. We focused on the effects of AMPK-mediated signaling and the NF-*κ*B pathway as well as the MAPK-related signaling in cholestatic liver diseases and evaluated the protective role of AICAR against hepatic injury and fibrosis triggered by cholestasis-related inflammation.

## 2. Materials and Methods

### 2.1. Animals

Twenty-four male Sprague Dawley (SD) rats aged 4–6 weeks (body weight 150–200 grams) were used for the current study. Rats were housed under conditions of constant ambient temperature (22°C), humidity, and a 12 h light/dark cycle. Rats were fed a commercial diet (Allied Foods). This study was approved by the Laboratory Animal Care Committee of Xi'an Jiaotong University.

### 2.2. Bile Duct Ligation

After 7 days, rats were evenly assigned into three groups: Sham-operated group, BDL group, and BDL+AICAR group. Sham-operated rats were subjected to a sham surgery, with no ligation of the bile duct. For BDL and BDL+AICAR rats, full surgical procedures were performed under isoflurane anaesthesia (2% isoflurane with 0.4 L/min O2 flow). Briefly, double ligation at the common bile duct and complete cutting at midpoint was conducted. The three groups of operation rats were maintained on a standard rat commercial diet and water ad libitum.

### 2.3. AICAR Administration

Two weeks after surgery, rats in the BDL+AICAR group started receiving AICAR treatment for 2 weeks by subcutaneous injection (160 mg/kg·day, diluted in 0.5 ml saline). Rats in the BDL group received 0.5 ml saline subcutaneous injection at the same time.

### 2.4. Serum and Liver Tissue Preparation

After 4 weeks of BDL, rats were anaesthetized by inhalation of diethyl ether, and subsequently anaesthesia was maintained with an intraperitoneal injection of ketamine (10 mg/100 g body weight) and xylazine (0.1 mg/100 mg body weight). Two millilitres of blood was drawn from the postcava. After centrifugation, the obtained serums were used for the detection of liver function and other biochemical indicators. All rats were sacrificed by haemospasia. Part of the liver tissues was harvested and fixed in 4% buffered formaldehyde and embedded in paraffin for histological analysis or snap frozen and stored at -80°C.

### 2.5. Histopathological Study

Liver histology was assessed on 5 *μ*m paraffin sections stained with haematoxylin and eosin (H&E) or sirius red staining. Images were taken with a Lecia DFC 490 digital camera. Results were confirmed by two individual observers.

### 2.6. Hydroxyproline Assay

The hydroxyproline assay was performed after 4 weeks of modelling in all three groups to determine collagen deposition in the liver (Hydroxyproline Assay Kit; Sigma-Aldrich). Briefly, samples were hydrolyzed at 120°C for 3h after bene tritum. The samples were centrifuged and the supernatant was transferred to 96-well plates. The assay reagents were subsequently added to the wells and incubated at 60°C for 90min. The hydroxyproline concentration was determined by comparing the absorbance of the samples to the standard curve at 560nm.

### 2.7. RNA Isolation and Quantitative Real-Time PCR (qPCR)

Extraction of total RNA from rat liver tissue was performed with the RNeasy kit (Qiagen, Valencia, CA) according to the manufacturer's instructions. cDNA was reverse transcribed from 1000 ng RNA using an Invitrogen Superscript First Strand Synthesis system. Quantification of mRNA expression was performed by quantitative real-time PCR (qPCR) using a Step One Plus qPCR platform (Applied Biosystems). The forward and reverse primers' sequences for all genes are listed in Table 1.

### 2.8. Western Immunoblot Analysis

Frozen liver tissue was pulverized and dissolved in an extraction buffer [50 mM Tris-HCl (pH 7.4), 1 mM EDTA, 1% Sodium Deoxycholate, 1% Triton X, and 150 mM NaCl] and lysed on ice for 1 h, then centrifuged at 14,000 rpm for 15 min, 4°C. Twenty micrograms of solubilised liver-tissue extracts was analysed on 10% SDS-PAGE gels. Gels were electroblotted onto a Hybond-P Extra nitrocellulose membrane (Amersham Biosciences) and blocked for 1 h at 22°C with TBST containing 5% skim milk powder. After blocking, the membrane was probed with following antibodies (AMPK, I*κ*B, P100, P52, STAT3, ERK, and JNK; Cell Signaling Technology, Inc.) in TBST containing 5% bovine serum Albumin overnight at 4°C, followed by secondary antibody (antirabbit or antimouse; Sigma) for 1 h at 22°C. Immunoreactive bands were detected by chemiluminescence (Super Signal West Femto and Pico; Thermo Scientific). The membranes were also probed with GAPDH antibody for loading control.

### 2.9. Data Analysis and Statistics

Quantitative data were expressed as mean ± standard error of STDEV/SQRT. Statistical analyses between control and treatment groups were performed using Student's t-test with SPSS 19.0. A P value of <0.05 was considered to be statistically significant.

## 3. Results

### 3.1. AICAR Histologically Improves Liver Inflammation and Fibrosis

**Figure 1:**
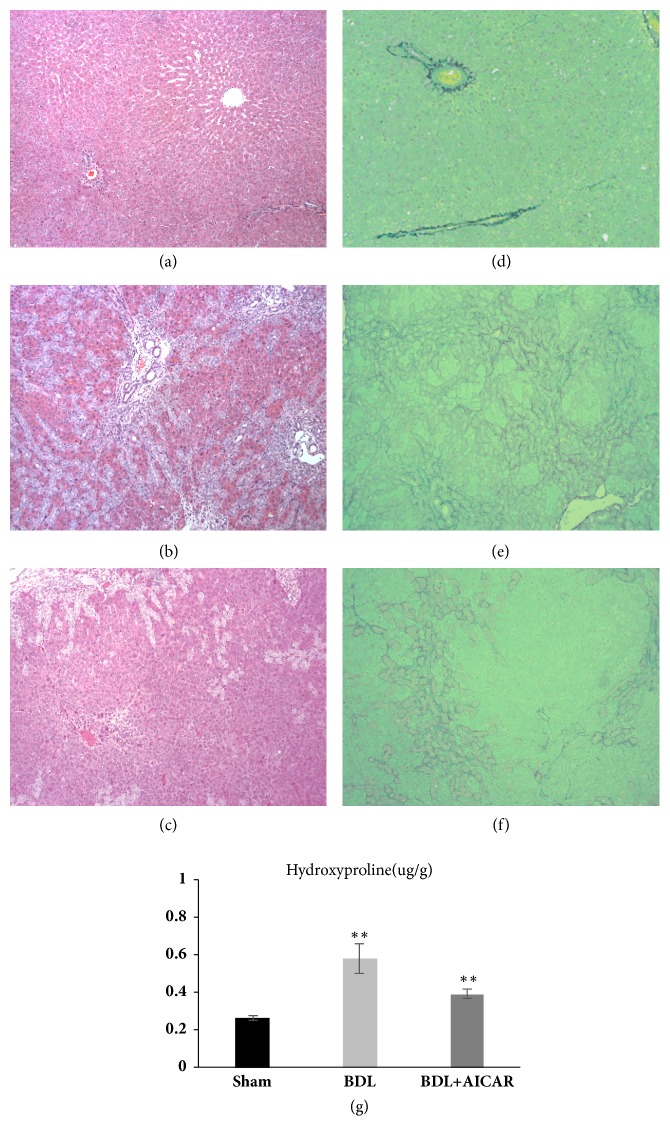
Hematoxylin & eosin (H&E), sirius red staining and hydroxyproline assay of rats liver tissue. Panels (a) and (d) show normal liver tissue of the H&E and Sirius red staining. Panels (b) and (e) represent BDL liver tissue with large areas of necrosis, inflammatory cell infiltration, and fibrosis. Newly formed bile ducts were also observed in BDL liver. Panels (c) and (f) represent less inflammatory cell infiltration and a decreased number of newly formed bile ducts in the BDL rats with the application of AICAR. Panel (g) shows the hydroxyproline content in three groups. *∗∗* P value <0.05, which was considered to be statistically significant in three groups.

Twenty animals survived the duration of the experimental period, and the rats which were subjected to BDL exhibited jaundice and weight loss (Sham group: 353 ± 22.5g, BDL group: 314 ± 18.5g, BDL+AICAR group: 321 ± 23.8g, and P < 0.01). Liver histological results showed a normal liver histological structure in the sham-operated group (Figure 1(a)), whereas there were large areas of parenchymal necrosis and inflammatory cell infiltration in BDL rats (Figure 1(b)). Moreover, multiple numbers of newly formed bile ducts were observed in the portal area, which extended to the adjacent hepatic parenchyma. Sirius red staining appeared normal in sham-operated rats (Figure 1(d)) and showed extensive regions of fibrosis in the BDL rats (Figure 1(e)). The livers of the BDL+AICAR group showed improvement in the histological findings, exhibiting a decreased number of newly formed bile ducts and less inflammatory cell infiltration (Figures 1(c) and 1(f)). BDL rats significantly increased hydroxyproline content compared with sham group (P < 0.01). Administration of AICAR attenuated the degree of hepatic fibrosis as indicated by the decrease of hydroxyproline content in BDL+AICAR group (P < 0.01) (Figure 1(g)).

### 3.2. AICAR Could Slightly Improve the Damaged Liver Function

**Figure 2:**
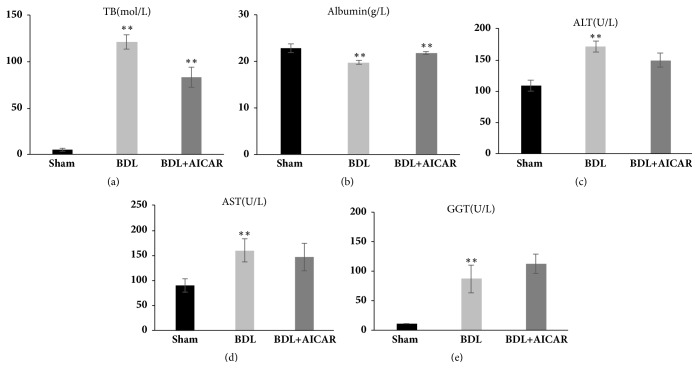
Liver function detected in sham operation, BDL, and BDL+AICAR groups. Panels (a)–(e) represent total bilirubin (TB), Albumin, Alanine Aminotransferase (ALT), Aspartate Aminotransferase (AST), and Glutamyl Transpeptidase (GGT), respectively. *∗∗* P value <0.05, which was considered to be statistically significant in three groups.

The liver function of BDL rats was severely damaged. Total bilirubin (TB) (25.3-fold, P < 0.01), Alanine Aminotransferase (ALT) (1.6-fold, P < 0.01), Aspartate Aminotransferase (AST) (1.7-fold, P < 0.05), Glutamyl Transpeptidase (GGT) (8.2-fold, P < 0.01), and Albumin (ALB) (0.7-fold, P < 0.05) were significantly increased compared with the sham-operated rats. After the administration of AICAR to BDL rats, the aforementioned biological indicators had been slightly recovered, with TB and ALB decreasing by 0.5- and 1.3-fold, respectively, while AST, ALT, and GGT showed no obvious changes (Figures 2(a)–2(e)).

### 3.3. The Changes of Related Inflammatory Cytokines and Target Genes in the BDL Rat Model

**Figure 3:**
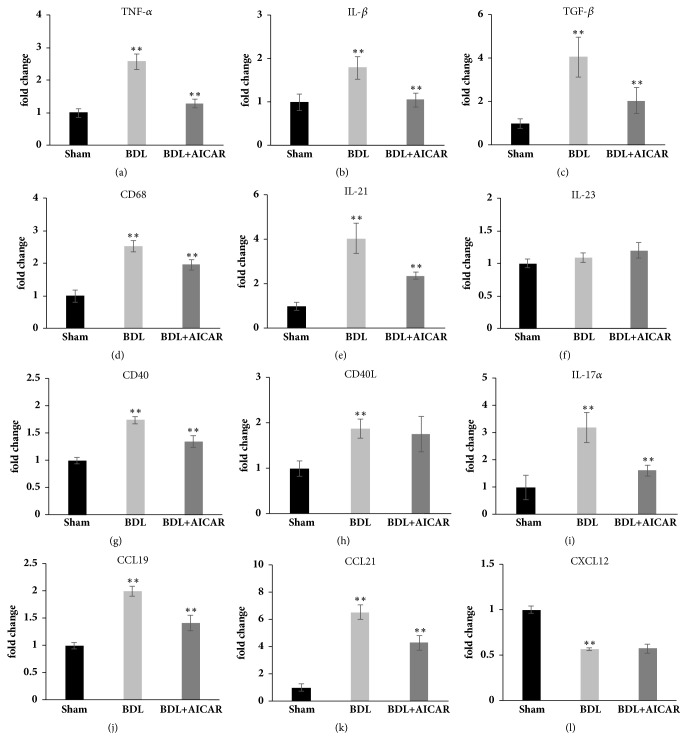
Real-time PCR results from rat liver tissue in sham operation, BDL, and BDL+AICAR groups. Panels (a)–(l) represent TNF-*α*, IL-1*β*, TGF-*β*, CD68, IL21, IL23, CD40, CD40Ligand, IL-17a, CCL21, CCL19, and CXCL12. *∗∗* P value <0.05, which was considered to be statistically significant in three groups.

The mRNA expression of canonical NF-*κ*B inflammatory cytokines, such as TNF-*α* (2.6-fold, P < 0.01), IL-1*β* (1.8-fold, P < 0.05), and TGF-*β* (4-fold, P < 0.01), was increased in the BDL rats compared with the sham rats (Figures 3(a)–3(c)). Moreover, the noncanonical NF-*κ*B specific target genes, such as CCL19 (2.0-fold, P < 0.01) and CCL21 (6.5-fold, P < 0.01), were also increased in BDL rats (Figures 3(j) and 3(k)). CD68, a macrophage inflammatory marker, was increased by 2.5-fold in BDL rats (P < 0.01), and there was an evident upregulation in members of the tumour necrosis factor superfamily genes such as CD40 (1.7-fold, P < 0.01) and CD40 ligand (1.9-fold, P < 0.01) in the liver of BDL rats (Figures 3(d), 3(g), and 3(h)). Additionally, T helper 17 effector cell cytokines, such as IL17a (3.2-fold, P < 0.01) and IL-21 (4.1-fold, P < 0.01), increased in BDL rat livers (Figures 3(i) and 3(e)). Further, IL-23 expression showed no change in the three investigated groups (P > 0.05) (Figure 3(f)).

### 3.4. AICAR via Activating AMPK Inhibits the NF-*κ*B Pathway to Attenuate Liver Injury and Fibrosis in BDL Rats

**Figure 4:**
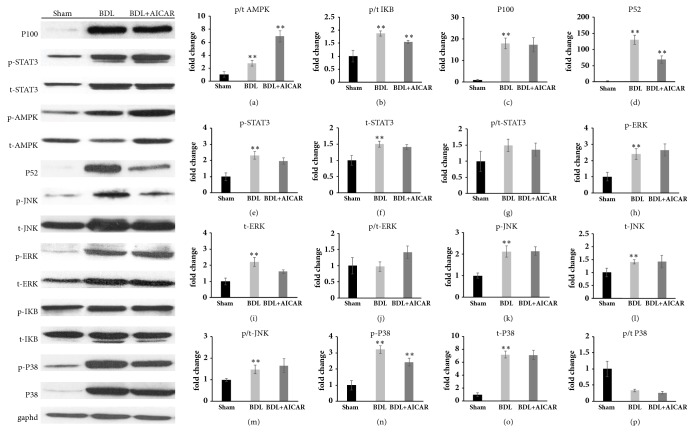
Representative Western blot showing levels of AMPK, I*κ*B, P100, p52, STAT3, ERK, JUK, and P38 protein in sham operation, BDL, and BDL+AICAR groups. *∗∗* P value <0.05 was considered to be statistically significant in three groups.

As NF-*κ*B activation can be induced through canonical and noncanonical pathways, thus I*κ*B, P100, and P52 expression were tested to evaluate the activity of these pathways, respectively. The results showed that the P-AMPK/AMPK ratio and P-I*κ*B/I*κ*B ratio increased by 2.6-fold (P < 0.05) and 2-fold (P < 0.05) and the P100 (18-fold, P < 0.01) and P52 (130-fold, P < 0.01) levels increased dramatically in BDL rats. For the BDL+AICAR rats, the P-AMPK/AMPK ratio increased by 2.56-fold but the P-I*κ*B/I*κ*B ratio (0.84-fold, P < 0.05) and P52 (0.5-fold, P < 0.01) decreased evidently compared with BDL rats, while P100 remained constant (Figures 4(a)–4(d)). Furthermore, compared with the BDL rats, TNF-*α* (0.5-fold, P < 0.01), IL-1*β* (0.6-fold, P < 0.05), and TGF-*β* (0.51-fold, P < 0.01) decreased in the BDL+AICAR rats, and there was a significant decline in CD68 (0.78-fold, P < 0.05), CD40 (0.78-fold, P < 0.05), IL17*α*(0.5-fold, P < 0.05), IL-21 (0.6-fold, P < 0.05), and CCL19 (0.7-fold, P < 0.01), while CD40 ligand and CCL21 remained unchanged (Figure 3).

As the Signal Transducer and Activator of Transcription 3 (STAT3) pathway has widely been reported to modulate inflammation and proliferation, we further noted that STAT3 phosphorylation was increased 2.3-fold in BDL rats compared with the sham rats (P < 0.01) and total STAT3 protein levels increased by 1.5-fold (P < 0.05). For the BDL+AICAR rats, it seemed there was no change in the expression of STAT3 compared with BDL rats (Figures 4(e)–4(g)). The MAPK family has also been verified to be closely related to the proliferation and activation of the hepatic stellate cell. We found that phosphor-ERK, phosphor-JNK, and p38 MAPK were increased by 2.4-, 2.1-, and 3.2-fold in BDL rats compared with the sham-operated rats (P < 0.01), while there was no statistical significance in the phosphor/total ratio expression of these indicators in BDL+AICAR rats (Figures 4(h)–4(p)).

## 4. Discussion

NF-*κ*B is a ubiquitous transcription factor that is activated by a variety of cytokines and mitogens and is thought to be a key regulator of genes involved in inflammation, responses to infection, and stress [18]. The signaling pathways that mediate NF-*κ*B activation can be classified into canonical and noncanonical pathways. In the present study, we found high expression of P-I*κ*B/I*κ*B ratio, P100, and p52, the important signaling proteins of canonical and noncanonical pathways, in BDL rats. Additionally, specific noncanonical NF-*κ*B target genes CCL19 and CCL21 had a higher expression in BDL rats of this study, indicating that the both canonical and noncanonical NF-*κ*B pathways play an important role in the occurrence of chronic cholestatic diseases.

Recently, AMPK has been reported as an anti-inflammatory protein which plays an important role in the regulation of cellular energy homeostasis [19], and its natural ligand AICAR exhibits inhibitory effects on immune responses in many animal models of inflammation [20]. The present study showed that NF-*κ*B signaling mediates liver injury and fibrosis in BDL mice, since it has been indicated that AMPK and NF-*κ*B signaling possess a close relationship in liver injury [21]. Therefore, AICAR was used to activate the AMPK to explore the underlying mechanism of AMPK and NF-*κ*B in liver injury and repair in the study. Meaningfully, after the administration of AICAR to BDL rats, the mRNA expression results showed the NF-kB inflammatory cytokines such as TNF-*α*, IL-1*β*, and TGF-*β* decreased evidently compared with BDL rats. Additionally, the protein P52 and specific target genes CCL19 (2.0-fold, P < 0.01) and CCL21 of noncanonical NF-*κ*B decreased evidently compared with BDL rats, suggesting that the NF-*κ*B pathway (more likely to be noncanonical) is inhibited and may be related to these classical pathway-related inflammatory factors. These findings indicate that AICAR-activated AMPK signaling in liver cells could inhibit the NF-*κ*B, mainly the noncanonical pathway, to relieve liver inflammation and fibrosis, which may be beneficial in treating liver injury or liver cirrhosis triggered by cholestatic liver disease. A similar study reported that curcumin increased the expression of AMPK and diminished NF-*κ*B protein in diabetic mice livers to relieve liver dysfunction [22], and it has been indicated that AICAR-induced activation of AMPK could ameliorate the hepatic steatosis and hypercholesterolemia associated with high TSH levels in patients with subclinical hypothyroidism [23]. Moreover, histological findings in AICAR+BDL rats showed a decreased number of newly formed bile ducts, less inflammatory cell infiltration, and that biological indicators such as TB and ALB had been slightly recovered compared with BDL rats, which further demonstrated that AICAR offers injured livers significant benefits at the cellular level.

Previous study showed that MAPK signaling pathway is involved in the formation of liver fibrosis through the regulation of hepatic stellate cell (HSC) activation, proliferation, and apoptosis. The extracellular signal regulated kinase (ERK), c-Jun N-terminal kinase (JNK), and p38 are important members of MAPK family [24]. Further, the different stimulatory factors acting on the relevant receptors of HSC can activate the signaling pathways of MAPK [25]. It has been reported that MAPK and NF-*κ*B pathways had a synergistic effect on CCL4-induced liver fibrosis [26]. Also, research has shown that AMPK activation with AICAR inhibited growth and proliferation in cardiac fibroblasts, which are involved inhibitory interactions between ERK and AMPK [27]. Our study found that the expression of the phosphor-ERK, phosphor-p38, and phosphor-JNK was significantly increased in the BDL rats but except for the expression of the P-JNK/JUK ratio was increased by 1.6-fold in BDL rats compared with the sham rats. There is no significant change of the three kinases after the administration of AICAR in our study, suggesting that the MAPK pathway probably may not exert a role in liver damage caused by BDL in the response to AICAR treatment, which is specific for AMPK. Furthermore, it may be attributed to the complicated process between the activation of HSC and the formation of hepatic fibrosis, as it involves multiple signaling pathways and cellular factors. Therefore, the mechanisms involved in the activation of AMPK to reduce liver injury may be complicated and further investigations are required to explain more specific mechanisms.

It is well known that macrophages and HSC play a pivotal role in the inflammatory reaction and liver fobrosis in cholestatic liver diseases and that macrophage infiltration can be induced by HSC to expand inflammatory effects. Our results showed that mRNA expressions of macrophage marker CD68 and inflammatory cytokines such as TNF-*α* and IL-1*β* were elevated in the livers of BDL rats, which is related to serious liver necrosis, inflammatory cell infiltration, and fibrosis in BDL rats (Figure 1). Furthermore, the inflammatory cytokines TNF-*α*, IL-1*β*, and TGF-*β*, as well as CD40, IL17a, IL-21, and CCL19, were closely related to the canonical and noncanonical NF-*κ*B pathway. After administration with AICAR, there was a significant decline in CD68 expression as well as in those related inflammatory cytokines. Histopathologically, the injured liver showed marked improvement and recovery. These findings indicate that AICAR can reduce liver injury by inhibiting the inflammatory reactions associated with the various pathways mentioned above. Moreover, recent studies have shown widespread expression of CD40 for inflammatory liver diseases, including allograft rejection, autoimmune disease, and viral hepatitis [28, 29]. Similarly, we found an enhanced expression of CD40 and CD40L in the liver of BDL rats and a reduced CD40 in BDL+ AICAR rats, prompting B cell-associated inflammatory responses, which are also involved in liver injury in the BDL model and are relieved in BDL+AICAR rats.

Another inflammatory cytokine, IL-17, which is closely related to T cells, mainly from T helper 17 cells, plays an important role in the chronic inflammation in liver diseases [30]. Studies have suggested that IL-17 stimulate Kupffer cells to express inflammatory cytokines like IL-1*β*, TNF-*α*, IL-6, and profibrogenic cytokine TGF-*β* in BDL rats [31]. Our study confirmed an increase expression of IL-17a mRNA in the BDL rat model. Additionally, IL-17 can directly stimulate collagen production in HSCs through STAT3 activation [31], and STAT3 is also suggested to play a protective role in the liver by enhancing hepatocyte regeneration [32]. We found an increased expression of phosphor-STAT3 in BDL rats, but P-STAT3/STAT3 ratio remained unchanged in the three groups, which suggests that STAT3 may not directly respond to cholestasis-related liver injury as well as enhancing fibrosis.

Recently, the effect of the noncanonical NF-*κ*B signaling has received much more attention in inflammation studies, many of which have indicated that the noncanonical NF-*κ*B pathway regulates the pathological effect function of Th17 cells and recalls responses of autoreactive T cells [33, 34], resulting in aberrant chemokine production and inflammatory cell recruitment and causing excessive chronic tissue damage and inflammation [35]. Our experimental results showed that, with the activator of AMPK as AICAR, liver injury inflammation can be eased through the inhibition of the noncanonical NF-*κ*B pathway, suggesting that AMPK is expected to become a new target for drug intervention for cholestatic diseases and that AICAR could play a protective role in BDL-induced liver injury and fibrosis. AICAR-mediated anti-inflammatory effects through the AMPK signaling are well known, but there has been less research on AICAR in inflammatory-related liver diseases such as PBC and PSC. A similar study confirmed that AICAR treatment could attenuate LPS-induced ROS-NF*κ*B signaling, immune responses, and liver injury in mouse models [21]. Another study of retinal degeneration found that AMPK activated by AICAR with a neuroprotective during retinal inflammation and the subsequent function of inhibiting NF-*κ*B possesses a protective effect on visual function [36]. Therefore, strategies of AICAR to activate AMPK signaling may provide alternatives to the current clinical approaches to inhibit inflammation and immune responses of liver diseases. However, the exact mechanisms of AICAR in hepatic fibrosis need to be further studied with much more specific experimental interventions.

In conclusion, our results showed that hepatocyte and bile duct injury in the BDL model of hepatic cholestasis activated the NF-*κ*B pathways, leading to persistent inflammatory responses. Moreover, the treatment of BDL-induced liver fibrosis with AICAR, activator of AMPK could inhibit the noncanonical NF-*κ*B pathways as well as the related inflammatory response to protect the liver function, suggesting that AMPK is expected to become a new target for drug intervention for inflammatory-related liver disease and that AICAR may be a potential therapy to prevent liver injury from stress or inflammation.

## Figures and Tables

**Table 1:** Forward and reverse primer sequences for each gene.

**GENEs**	**Forward (F) and Reverse (R) Primer sequences**
**TNF-**α	(F) TCT CTA ATC AGC CCT CTG GCC CAG G
	(R) TAC AAC ATG GGC TAC AGG CTT GTC AC

**TGF-**β	(F) TCG ACA TGG AGC TGG TGA AA
	(R) GAG CCT TAG TTT GGA CAG GAT CTG

**IL-1**β	(F) TGTGATGAAAGACGGCACAC
	(R) CTTCTTCTTTGGGTATTGTTTGG

**IL-17**α	(F) CTT CAC CCT GGA CTC TGA GC
	(R) CCT CAG CGT TGA CAC AGC

**IL-21**	(F) CAA AGG CCA GAT CAC CTT CTG
	(R) GCC CCT TTA CAT CTT GTG GA

**IL-23**	(F) TGT GGA CCA GCT TCA TAC CTC
	(R) TCT CCC AGT GGT GAT CCT CT

**CD68**	(F) TCT GAC CTT GCT GGT ACT GC
	(R) GAA GAG TGG CAG CCT TTT TG

**CCL19**	(F)CCCGTGTGACCCCACTACT
	(R)GTCTTCCGCATCGTTAGCAC

**CCL21**	(F)CCATCCCAGCAATCCTGTT
	(R)GGCTTCCTCAGGGTTTGC

**CD40**	(F)CATTCCCGTCGTGATGGT
	(R) GCCTCAGGGGGTAAGACC

**CD40L**	(F) TGAGGCCAACAGTAATGCAG
	(R)GAAGGTGACTTGGGTGTAGACAT

## Data Availability

The technical appendix, statistical code, and dataset are available from the corresponding author at huliangshuo1983@hotmail.com.
